# Metabolic Alterations Associated with γ-Hydroxybutyric Acid and the Potential of Metabolites as Biomarkers of Its Exposure

**DOI:** 10.3390/metabo11020101

**Published:** 2021-02-10

**Authors:** Suryun Jung, Suji Kim, Yujin Seo, Sooyeun Lee

**Affiliations:** 1 College of Pharmacy, Keimyung University, 1095 Dalgubeoldaero, Dalseo-gu, Daegu 42601, Korea; susu73@kmu.ac.kr (S.J.); kimsuji921@naver.com (S.K.); syj653@naver.com (Y.S.); 2 Center for Forensic Pharmaceutical Science, Keimyung University, 1095 Dalgubeoldaero, Dalseo-gu, Daegu 42601, Korea

**Keywords:** γ-hydroxybutyrate, drugs of abuse, drug-facilitated crimes, metabolomics

## Abstract

γ-Hydroxybutyric acid (GHB) is an endogenous short chain fatty acid that acts as a neurotransmitter and neuromodulator in the mammalian brain. It has often been illegally abused or misused due to its strong anesthetic effect, particularly in drug-facilitated crimes worldwide. However, proving its ingestion is not straightforward because of the difficulty in distinguishing between endogenous and exogenous GHB, as well as its rapid metabolism. Metabolomics and metabolism studies have recently been used to identify potential biomarkers of GHB exposure. This mini-review provides an overview of GHB-associated metabolic alterations and explores the potential of metabolites for application as biomarkers of GHB exposure. For this, we discuss the biosynthesis and metabolism of GHB, analytical issues of GHB in biological samples, alterations in metabolic pathways, and changes in the levels of GHB conjugates in biological samples from animal and human studies. Metabolic alterations in organic acids, amino acids, and polyamines in urine enable discrimination between GHB-ingested animals or humans and controls. The potential of GHB conjugates has been investigated in a variety of clinical settings. Despite the recent growth in the application of metabolomics and metabolism studies associated with GHB exposure, it remains challenging to distinguish between endogenous and exogenous GHB. This review highlights the significance of further metabolomics and metabolism studies for the discovery of practical peripheral biomarkers of GHB exposure.

## 1. Introduction

Metabolomics is a field of omics science that investigates changes in metabolites with molecular weights of 1500 Da or less in biological samples and aims to understand metabolic pathways related to abnormal conditions or diseases. It is used to discover biomarkers that can serve as indicators of normal and pathological processes or reactions upon exposure to drugs, toxicants, or any intervention [1,2]. Analytical methods, including gas chromatography-mass spectrometry (GC-MS), liquid chromatography-mass spectrometry (LC-MS), nuclear magnetic resonance (NMR), and capillary electrophoresis-mass spectrometry (CE-MS), are used to investigate alterations in the concentrations of metabolites in a variety of biological samples such as blood, urine, and hair. In general, MS offers the advantage of displaying high sensitivity and a wide detection range, while NMR allows for non-destructive and minimal sample preparation [2,3]. Plasma and serum samples provide much information on physiological and pathological conditions in a particular biological system, over a short period of time [4]. Since many biogenic products eventually find their way to the urine, urinary metabolites are very beneficial for understanding the condition of diseases. Owing to the simple and noninvasive sampling process, the presence of metabolites in large quantities, and the extended detection window, urine is considered an ideal sample compared to plasma and serum for biomarker monitoring in clinical analyses [5,6]. Among biological samples, hair is more recently being used to monitor chronic drug use or chronic diseases, because it provides long detection windows, and segmental analysis of hair reflects the toxicological or pathological history [7]. Analytical approaches for metabolomics include targeted and untargeted analyses. Targeted analysis investigates metabolic changes based on physicochemically similar metabolomes (e.g., carbohydrates, amino acids (AAs), organic acids (OAs), nucleosides) or biochemical (e.g., glycolysis, gluconeogenesis, β-oxidation, or citric acid cycle) and metabolic (e.g., phase-I and -II metabolism) pathways. Therefore, selective and sensitive sample preparation methods and optimized analytical techniques are necessary for evaluating selected metabolites. Untargeted analysis is a method for analyzing the overall metabolic change in a selected biological sample, based on extensive information on unknown features, followed by the assignment of significantly altered features to specific metabolites. To investigate the maximum number of metabolites possible, non-selective approaches have been adopted for sample preparation and instrumental analysis [2,4]. In particular, metabolomics is currently being used to identify endogenous metabolites generated after exposure to addictive drugs [8,9,10,11]. This metabolomics approach can provide a new foundation for identifying effective diagnostic markers or therapeutic targets, if the knowledge of the mechanisms of a drug’s pharmacodynamic or pharmacokinetic properties is limited [12].

γ-Hydroxybutyric acid (4-hydroxybutyric acid, GHB, m.w. 104.1 g/mol) is a naturally occurring short-chain fatty acid in the human brain that acts as a neurotransmitter and neuromodulator [13]. GHB was developed as an anesthetic in the 1960s; however, its use was limited thereafter due to the occurrence of pain and delirium [14]. It is currently being used to treat Excessive Daytime Sleepiness (EDS) and cataplexy (Xyrem^®^; sodium oxybate) in patients with narcolepsy—with doses ranging from 3.0 to 9.0 g and treatment lasting from 4 to 8 weeks [15]—and for alcohol dependence and withdrawal (Alcover^®^) in Austria and Italy. However, as with other psychotropic drugs, there is a high risk of side effects upon GHB abuse, so its use other than for treatment of disease is strictly prohibited. Ingestion of GHB in recreational doses (>10 mg/kg) relieves tension, induces euphoria, and increases sexual pleasure. However, ingestion of 20–30 mg/kg of GHB can lead to dizziness, drowsiness, nausea, and vomiting, while intake of GHB over 50 mg/kg can lead to coma and death [16,17]. Moreover, people intoxicated with GHB may suffer from anterograde amnesia, which is one of the reasons criminals use this drug in sexual assault cases [18]. Possible withdrawal symptoms post GHB use include mild tremor, tachycardia, high blood pressure, anxiety, agitation, seizures, insomnia, severe disorientation, hallucinations, delirium, and rhabdomyolysis [19]. The average self-administration dose reported in dependent patients ranged from 32 to 67.2 g/day [20] to a maximum of 144 g/day [21] at 45-min to 2.5-h intervals. The severity of physical dependence on GHB is affected by the dose and duration of abuse [22], and high doses and/or long-term administration may be important factors influencing the physical dependence on GHB [23]. To date, a number of studies have been conducted to develop a therapeutic agent to reverse GHB-induced intoxication and sedative effects. Representatively, physostigmine has shown the possibility of recovering the GHB-induced change in consciousness state [24,25]. However, physostigmine increases cardiovascular-related side effects [26], while naloxone [27], an opiate antagonist, and flumazenil [28], a selective benzodiazepine receptor antagonist, have not been found to be effective in reversing the sedative effects of GHB. Upon studying whether the γ-aminobutyric acid (GABA)-B receptor antagonist has an effect on reducing mortality due to excessive GHB intake in mice, no significant effect was found [29]. Taken together, there is no antidote for GHB intoxication; in addition, the gap between the recreational and lethal doses of GHB is narrow, resulting in frequent accidental overdose deaths [30,31]. Therefore, various studies need to be carried out in clinical and forensic areas to diagnose and treat GHB poisoning and addiction. This review summarizes the methods and results of such studies, while focusing on studies of exogenous GHB (ExGHB) exploration using metabolomics or metabolite analysis, in addition to providing the latest information on the biomarkers of ExGHB. With specific focus on research publications from January 2010 till September 2020, a search was conducted in PubMed using the following keywords: [“γ-hydroxybutyric acid,” “γ-hydroxybutyrate,” or “GHB”] and [“metabolomics” or “metabolites”]. A total of 222 papers were searched with the keywords, and 25 papers were selected, excluding those with duplicate and low accuracy. Among them, 10 articles [32,33,34,35,36,37,38,39,40,41] were selected and analyzed after the exclusion of 14 papers. The exclusion criteria were as follows: reviews (*n* = 2) and purpose different from that of the current study (e.g., plant, succinic semialdehyde dehydrogenase deficiency, among others; *n* = 12). Additionally, one article [42] found in the reference list in one [34] of the searched articles was included since it is a metabolomics study of GHB.

## 2. Biosynthesis and Metabolism of GHB

**Figure 1:**
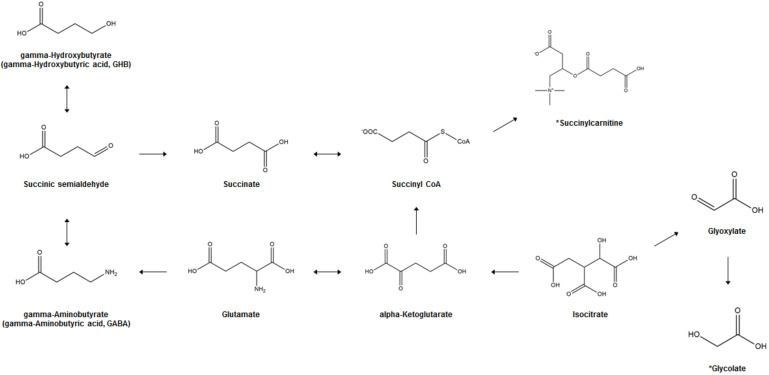
GHB biosynthesis and metabolism and potential biomarkers of GHB exposure. *; Proposed potential biomarker.

Figure 1 illustrates the biosynthesis and metabolism of GHB and the potential bi-omarkers for GHB exposure. In the human brain, GHB (endogenous GHB; EnGHB) is produced from GABA by the action of GABA aminotransferase and succinic semialdehyde reductase. EnGHB concentration is the highest in the striatum, about 11–25 μM [43], and the lowest in certain areas of the cerebellum and cerebral cortex [44]. It is also found in the heart, liver, kidney, muscle, and brown fat, but its function in these organs is still unclear [45]. The generated GHB is converted to succinic semialdehyde (SSA) and converted back to GABA [46], or is metabolized to succinic acid and removed for use in energy metabolism through the Krebs cycle [47]. In addition, a small amount of GHB that is not metabolized is excreted in the urine [17]. Since GHB exists as a physiological compound in the human body, and ExGHB exhibits the same physiological effects using almost the same neurobiological pathway as EnGHB, it is difficult to distinguish between EnGHB and ExGHB [48,49].

Another characteristic of GHB is that its metabolism occurs very quickly. A single oral intake (25 mg/kg body weight) of GHB in healthy adults results in the maximum plasma concentration within 30–90 min, with an average half-life of 40–60 min in the plasma [50]. GHB can be detected only within 6 h in the blood [51] and 12 h in the urine [52]. Because of these metabolic properties of GHB, it is very difficult to detect GHB in human biological samples to prove abuse or drug-related crime [53]. Until now, a number of studies have suggested cut-offs for the distinction of EnGHB and ExGHB concentrations in the urine or serum/plasma, but no consensus has been reached. Currently, 6–10 mg/L in urine and at least 4 mg/L in blood are accepted as typical human ExGHB cut-off concentrations [54,55,56]. However, some studies suggest a cut-off concentration of 1 mg/L in blood [57] and 5 mg/L [58,59] or 2 mg/L [60] in urine. Andresen et al. [61] collected blood and urine samples from 50 subjects who had never ingested GHB and analyzed them using GC-MS. The concentration of GHB ranged from 0.62–3.24 mg/L (mean = 1.14 mg/L and median = 0.97 mg/L) in serum and 0.64–4.20 mg/L (mean = 1.21 mg/L, median = 0.96 mg/L) in urine. Based on these results, it was suggested that the use of 6 mg/L for the urine GHB cut-off value instead of 10 mg/L is appropriate to avoid false negative interpretation. In addition, Kang et al. [62] reported that 0.09–1.8 g/mL of GHB was detected in the urine of 79 healthy volunteers, and when this concentration was adjusted to the creatine concentration, 4.5–530 g/mmol of GHB was present. Sex and age, but not smoking, alcohol, or caffeine intake, have been reported to have an effect on the concentration of EnGHB in urine. Despite the fact that multiple studies and discussions have been published on the cut-off level for GHB detection in biological specimens, the application of these methods to forensic cases is not simple. There are numerous factors to be considered, such as the in vivo and in vitro production of GHB in human biological specimens depending on the post-mortem interval, the time between sampling and analysis, the sample storage conditions (e.g., storage temperature, storage period, and addition of preservatives, among others), and the concomitant use of GHB with other drugs, including ethanol [63,64]. Although unusual, the conditions of GHBuria (γ-Hydroxybutyric aciduria) and succinic semialdehyde dehydrogenase deficiency (SSADH-D) should also be considered during diagnosis. SSADH-D is a disease caused by a mitochondrial enzyme that results in abnormal metabolism of the neurotransmitter GABA, leading to accumulation of GABA and GHB in the body, while GHBuria is a rare genetic disease characterized by the excretion of accumulated excess GHB in the urine [65]. Increased endogenous GABA and GHB levels through SSADH-D contribute to neurological disorders such as cognitive deficit and speech impairment, ataxia, hypotension, decreased reflexes, behavioral dysregulation, and compulsion [66].

## 3. Analytical Issues of GHB in Biological Samples

For forensic toxicological purposes in drug-facilitated crimes or post-mortem cases, biological samples such as urine [55,58,67,68], saliva [69,70], vitreous humor [71,72,73], blood spots [74,75], and hair [76,77,78] have often been analyzed using GC-MS or LC-MS, with or without chemical derivatization. However, the accurate quantification of GHB in biological samples is complicated because of the absence of an analyte-free matrix and the endogenous presence of GHB isomers, such as beta-hydroxybutyrate and alpha-hydroxybutyrate, the concentrations of which increase in diabetics [79]. In previous studies, quantification of urinary GHB was performed using water [68,80] or synthetic urine [81] as an analyte-free matrix and ^2^H_6_-GHB as a surrogate standard together with ^13^C_2_-dl-3-hydroxybutyrate as an internal standard [62]. Missing matrix-matched calibrators is considered complex because parallelism between the alternative analyte-free matrix and the authentic matrix or surrogate and original standards needs to be confirmed before their use.

Another critical issue in the analysis of GHB in biological samples is the in vitro production of GHB under sample storage conditions (e.g., temperature, period, addition of preservatives, etc.). Nevertheless, the stability of GHB has been only partially or not investigated during method validation in many previous studies [68,82,83,84]. In previous studies, EnGHB was stable in blood or urine at −20 °C with fluoride preservation, while an increase in GHB was observed at a higher temperature (4 °C) and/or without preservatives [67,85]. In another study, the concentration of EnGHB increased by greater than 2-fold at 4 °C for 7 or 14 days and to more than 110% at −20 °C for 7 or 14 days. EnGHB was stable only at −80 °C for 7 or 14 days [62].

## 4. GHB-Associated Metabolic Changes in AA, OA, and Polyamine (PA)

Table 1 summarizes metabolomics studies performed to investigate metabolic changes following GHB exposure in the urine of rats or humans [32,33,34,42]. GHB-associated metabolic changes in animal studies [32,34,42] were further confirmed in a clinical setting [33]. To perform the targeted metabolic profiling of AA, OA, and PA in rat urine, chemical derivatization was conducted for specific functional groups followed by GC-MS analysis. For untargeted metabolomics using NMR, human urine was lyophilized and reconstituted in D_2_O.

A previous study demonstrated the accumulation of D-2-hydroxyglutaric acid (D-2-HG) in blood and urine from baboons following GHB exposure, based on the hypothesis of conversion of GHB to D-2-HG, a reaction catalyzed by d-2-hydroxyglutarate transhydrogenase [86]. In the intermediate metabolic pathway, various OAs such as mono-, di-, and tri-carboxylic acids with hydroxyl, aromatic rings, and carbonyl groups as metabolites are involved in the metabolism of AAs as well as the tricarboxylic acid (TCA) cycle and β-oxidation of fatty acids. According to previous studies, changes in OA levels are reported in the conditions of diabetes [87], cancer [88] and genetic metabolic disorders [89,90]. An animal study was performed to study the effect of GHB administration for 1 or 10 days (600 mg/kg, i.p., once/day) on TCA cycle-related OA and D-2-HG levels in rats using GC-MS analysis. The results showed that single or multiple doses of GHB increased most of the OA, including GHB and D-2-HG, in the urine of rats (*p* < 0.05); in addition, the single dose group showed a greater increase in these factors than the multiple dose group. From these results, it was concluded that citrate, isocitrate, and cis-aconitate profiling are useful biomarkers for discriminating GHB intoxication [42]. AAs are precursors and products of OAs that are closely related to GHB metabolism and the TCA cycle; AA in the blood gets excreted into urine to maintain homeostasis. Therefore, profiling of AAs using urine samples is very useful for monitoring altered metabolism and understanding biochemical changes [90,91]. Seo et al. [34] profiled AAs, including GABA and glutamic acid, using GC-MS after GHB administration (600 mg/kg, i.p., once/day) to rats for 1 or 10 days. They found that 28 AAs were detected in urine in the control and GHB-intake groups at levels exceeding the limit of quantification of the AA profiling method. In addition, levels of GABA and glutamic acid, which are GHB metabolites, were significantly higher in the urine of the single treatment group. In addition, it has been reported that phenylalanine, glutamic acid, aspartic acid, asparagine, and methionine are metabolites that can help in distinguishing whether or not GHB has been taken and the number of doses. These results suggest that changes in AA metabolism could be used as a useful biomarker for discriminating GHB administration. PA is being studied with a focus on cell growth, cell proliferation, protein, and nucleic acid synthesis by controlling its acetylation-deacetylation, according to changes in biochemical conditions [92,93,94]. Recently, studies on PA have focused on its role in a PA-mediated stress response signaling system and as a marker for monitoring and diagnosing various disease states [95,96,97]. Therefore, endogenous PA profile analysis is important for understanding biochemical changes post GHB exposure. Accordingly, Lee et al. [32] administered GHB (600 mg/kg, i.p., once/day) to rats for 1 or 10 days and then profiled PA in the urine using GC-MS. The levels of N^1^-acetylspermine, putrescine, N^1^-acetylspermidine, and spermine were found to be significantly higher in the single administration group, as compared to the control group, but the levels of putrescine and N^1^-acetylspermidine were found to be significantly lower in the multiple administration group. In summary, there were significant differences in the levels of N^1^-acetylspermine between the three groups, while the levels of spermine differed significantly between the administered and non-administered groups. Therefore, N^1^-acetylspermine and spermine have been suggested as potential biomarkers of GHB exposure and poisoning.

Although little metabolomics studies have been conducted on clinical settings, the elevated urinary levels of OAs, such as succinate, following GHB exposure, were observed in a previous human study [33]. Palomino-Schätzlein et al. [33] collected urine samples for 30 h after one dose of GHB (Xyrem^®^, sodium oxybate, 500 mg/L) in healthy adult men and women and analyzed them using NMR spectroscopy. There was a significant increase in the concentration of succinate and glycolate in urine post GHB administration. However, while the levels of succinate decreased rapidly, glycolate was identified as a biomarker capable of discriminating ExGHB, as it maintained a high level for more than 20 h, as compared to the levels before GHB intake.

## 5. Changes in the Levels of GHB Conjugates upon GHB Exposure

In recent studies, GHB-GLUC and GHB-SUL, which are phase-II-metabolites of GHB, have been considered as potential biomarkers for GHB exposure despite controversial data on their merits, such as longer detection window, compared to GHB [35,36,98,99]. A number of studies have attempted to develop and verify methods for measuring phase-II-metabolites in various biological samples, including human urine and blood (Table 2; Table 3). To selectively and sensitively quantify GHB and/or GHB conjugates in a variety of samples, such as urine, blood, nail, and hair, different sample preparation approaches, including protein precipitation, chemical derivatization, and solid phase extraction, followed by GC-MS/MS or LC-MS/MS, have been employed. The baseline levels of phase-II-metabolites were measured in urine, nails, and hair in a healthy general population without GHB intake. GHB-GLUC was found to be in the range of 0.11–5.0 mg/mL in urine [36] and 0.08–0.252 ng/mg in nails [76]. As a result of measuring the EnGHB concentration in the hairs of 60 healthy adults and 5 infants, a GHB concentration of 0.11–0.96 ng/mg (average 0.38 ± 0.25 ng/mg) was detected in 44 samples. In addition, GHB concentrations between LOD and LOQ (0.033–0.1 ng/mg) were detected in 21 samples. However, GHB-GLUC was detected at concentrations between LOD and LOQ (0.11–0.37 ng/mg) in only three samples (4.6%) and was not detected in the remaining samples [37]. Upon measurement of the levels in a post-mortem peripheral blood sample, 0.8–23 mg/L of GHB was detected, whereas GHB-GLUC was not detected. Hanisch et al. [35] reported that 70–170 μg/mL of GHB-SUL was detected in human urine in their study.

Piper et al. [40] measured the concentrations of GHB-conjugate in the urine of athletes (*n* = 100) and physical college students (*n* = 50). GHB-SULF concentration was 9–11,500 ng/mL and GHB-GLUC was 500–19,800 ng/mL. They used these values to calculate a preliminary reference population-based threshold and compared it to the concentration measured 72 h post GHB administration in urine samples from three volunteers. The results showed that GHB-SULF and GHB-GLUC were not suitable for judging GHB exposure because of the large difference in concentrations between the individuals. However, in the case of GHB-GLUC, there was an improvement in the reference population-based threshold upon correcting for urine-specific gravity. Accordingly, it was found that the ratio of GHB-GLUC and β-citryl glutamic acid was a promising tool to extend the possibility of GHB detection, as it was found that β-citryl glutamic acid is another compound in urine that has a significant correlation with GHB-GLUC excretion. Steuer et al. [41] conducted a randomized, placebo-controlled crossover study of 20 healthy male volunteers. Analysis of urine samples obtained 4.5 h post GHB administration showed significantly increased concentrations of eight compounds, including the GHB conjugates GHB-carnitine, GHB-glutamate, and GHB-glycine, and endogenous compounds glycolate and succinylcarnitine. GHB-carnitine, GHB-glutamate, and GHB-glycine were detected only in the GHB intake group and not in the placebo group. However, glycolate and succinylcarnitine were detected in both the GHB-ingestion and placebo groups, but GHB exposure was proven by showing a significant difference in concentration.

## 6. Future Directions and Conclusions

**Figure 2:**
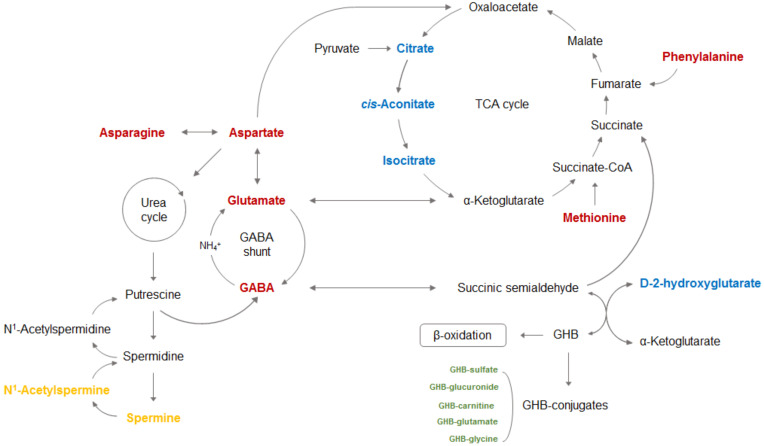
Altered metabolic pathway associated with GHB exposure based on findings from previous studies using rats and humans. Proposed biomarkers are displayed in the following colors (blue color, organic acid; red color, amino acid; yellow color, polyamine; green color, GHB-conjugate).

GHB exists as a physiological compound in the human body; it is difficult to distinguish between EnGHB and ExGHB. Because of its rapid metabolism, it is difficult to identify abuse or drug-related crimes by detecting GHB in human biological samples. Recently, a number of studies focusing on metabolites have been conducted in the field of forensic toxicology. Characterizing and quantifying metabolites in organisms has the advantage of not only being able to predict phenotypes but also suggesting potential biomarkers. Therefore, in this review, the study method and proposed metabolites were reviewed, focusing on the study of ExGHB detection using metabolites. Figure 2 shows a schematic representation of the proposed biomarkers identified in metabolomics and metabolism studies following GHB exposure in rats and humans. Despite possible gaps in the metabolic responses between rats and humans, the detection of metabolic alterations in urinary OA, AA, and PA can help distinguish between GHB-ingesting animals or humans and controls. The potential of GHB conjugates has been investigated in various clinical settings. In studies using rat urine, OA (citric acid, isocitric acid, cis-aconitic acid, D-2-HG), AA (phenylalanine, glutamic acid, aspartic acid, aspartic acid, methionine), and PA (N^1^-acetylspermine, spermine) were proposed as biomarkers to detect ExGHB. In addition, glycolic acid was also proposed as a biomarker in studies using human urine. However, there are many limitations in using these metabolites as biomarkers to determine ExGHB. Urine OA is affected by the subject’s health status, nutritional status, vitamin deficiency, and drugs. In particular, mitochondrial dysfunction, which is considered to be one of the causes of chronic disease, affects metabolites related to the TCA cycle. In addition, AA is closely related to prediabetes and insulin resistance, so it is suggested as a predictor of type 2 diabetes, while PA is a powerful metabolite that can predict Parkinson’s disease. Various other factors, such as physical activity, pharmacological treatment, nutritional interventions, and exposure to other substances also affect the levels of metabolites in biological samples [100]. Significant alterations in the levels of lipid-related metabolites [101,102,103,104,105,106], tryptophan and other amino acid-related metabolites, and tricarboxylic acid (TCA) cycle components, including malate, aconitate, citrate, fumarate, succinate, and alpha-ketoglutarate [106,107,108,109,110,111,112] were observed after endurance exercise.

Therefore, in order to use the metabolites related to the TCA cycle and metabolites related to the urea cycle as biomarkers for ExGHB identification, the changes in GHB metabolism, according to physiological conditions, must be identified. Meanwhile, there is increasing interest in phase-II-metabolites (GHB-GLUC and GHB-SUL), which have a relatively longer half-life and slower removal rate, as compared to GHB. However, there is insufficient evidence to be presented as a biomarker for discriminating GHB exposure, since most previous studies are in the stage of measuring the basal level of phase-II metabolites in urine, nails, and hair in healthy humans. Despite the recent growth in the application of metabolomics to understand the effect of GHB on the metabolic system, there are numerous opportunities for enhancing and expanding the impact of metabolomics in the discovery of biomarkers for GHB exposure. In particular, toxicokinetic investigations of metabolites or metabolite ratios need to be considered to overcome the rapid elimination of GHB. Moreover, GHB-related potential biomarkers proposed in previous studies need to be validated in clinical settings.

## Figures and Tables

**Table 1:** Summary of metabolic changes in organic acids, amino acids, and polyamines following GHB exposure.

Reference No.	Sample	Sample Preparation	Analytical Platform (Untargeted or Targeted)	Treatment (Administration Dose, Route, and No. of Doses)	Sampling Time	Metabolic Changes	Summary
[42]	Male SD rat (*n* = 18), urine	Methoxime/tert-butyldimethylsilyl derivatization	GC-SIM-MS(targeted)	600 mg/kg GHB, i.p. once per day for 10 days	For 12 h following single administration	Pyruvic acid (↑), acetoacetic acid (↓), lactic acid (↑), glycolic acid (↑), 2-hydroxybutyric acid (↓), malonic acid (↓), succinic acid (↑), fumaric acid (↑), oxaloacetic acid (↑), malic acid (↑), α-ketoglutaric acid (↑), 2-hydroxyglutaric acid (↑), cis-aconitic acid (↑), citric acid (↑), isocitric acid (↑), γ-hydroxybutyric acid (↑)	Alteration of organic acid metabolism related with tricarboxylic acid cycle Key metabolite: 2-hydroxyglutaric acid
					For 12 h following multiple administration (10 times)	Pyruvic acid (↑), acetoacetic acid (↑), lactic acid (↑), glycolic acid (↑), 2-hydroxybutyric acid (↑), malonic acid (↑), succinic acid (↑), fumaric acid (↑), oxaloacetic acid (↑), malic acid (↑), α-ketoglutaric acid (↑), 2-hydroxyglutaric acid (↑), cis-aconitic acid (↑), citric acid (↑), isocitric acid (↑), γ-hydroxybutyric acid (↑)	
[34]	Male SD rat (*n* = 25), urine	Ethoxycarbonyl/tert-butyldimethylsilyl derivatization	GC-SIM-MS(targeted)	600 mg/kg GHB, i.p. once per day for 10 days	For 12 h following single administration	Alanine (↑), glycine (↑), α-aminobutyric acid (↑), valine (↑), β-aminoisobutyric acid (↑), leucine, isoleucine (↑), serine (↑), proline (↑), γ-aminobutyric acid (↑), pipecolic acid (↑), 4-hydroxyproline (↑), methionine (↑), phenylalanine (↑), aspartic acid (↑), glutamic acid (↑), asparagine (↑), ornithine (↑), lysine (↑), histidine (↑), tyrosine (↑), tryptophan (↑), glutamine (↓)	Alteration of amino acid metabolism Key metabolite(s): phenylalanine, glutamic acid, aspartic acid, asparagine, and methionine
					For 12 h following multiple administration (10 times)	Leucine (↑), isoleucine (↑), serine (↑), proline (↑), histidine (↓), phenylalanine (↓), γ-aminobutyric acid (↑), pyroglutamic acid (↑), α-aminoadipic acid (↑), glycine (↓), methionine (↓), tyrosine (↓) 4-hydroxyproline (↓), aspartic acid (↓), glutamic acid (↓), asparagine (↓), ornithine (↓), glutamine (↓), lysine (↓)	
[32]	Male SD rat (*n* = 18), urine	N-ethoxylcarbonyl-N-pentafluoropropionyl derivatization	GC-SIM-MS(targeted)	600 mg/kg GHB, i.p. once per day for 10 days	For 12 h following single administration	Putrescine (↑), N^1^-acetylspermidine (↑), spermine (↑), N^1^-acetylspermine (↑)	Alteration of polyamine metabolism Key metabolite(s): *N*1-acetylspermine and spermine
					For 12 h following multiple administration (10 times)	Putrescine (↓), N^1^-acetylspermidine (↓), spermine (↑), N^1^-acetylspermine (↑)	
[33]	Healthy men & women (*n* = 12, each), urine	Lyophilization and reconstitution in D_2_O	NMR(untargeted)	25 mg/kg GHB (Xyrem^®^)	Urine: 10 min, 1, 2, 4, 6, 14, 20, 24, and 30 h post dose	Glycolate (↑), succinate (↑)	Confirmation of glycolate and succinate as potent markers for GHB, Slower elimination of glycolate (even after 24 h) than succinate (at time point of 6 h)

GC, gas chromatography; SIM, selected ion monitoring; MS, mass spectrometry; i.p., intraperitoneal injection; ↑, significantly increased vs. vehicle group; ↓, significantly decreased vs. vehicle group.

**Table 2:** Summary of analysis of GHB conjugates in human biological samples from non-GHB users.

Reference No.	Sample	Sample Preparation	Analytical Platform	Concentration Range
[36]	Anonymous clinical urine samples (*n* = 50)	Dilution	LC-MS/MS	GHB-GLUC, 0.11–5.0 mgL (mean, 1.3 ± 1.2 mgL).
[35]	Authentic urine samples (*n* = 5)	Protein precipitation	LC-MS/MS	GHB-SUL, 70–170 mgL
[38]	Post-mortem peripheral blood samples (*n* = 12)	Protein precipitation followed by trimethylsilyl derivatization	GC-MS/MS	GHB, 0.8–23 mg/L; GHB-GLUC, ND
[39]	Healthy adults & children nail samples (*n* = 90)	Digestion followed by solid phase extraction	LC-MS/MS	GHB, 0.3–3.8 ng/mg in fingernails and 0.3–2.4 ng/mg in toenails; GHB-GLUC, 0.08–0.252 ng/mg in fingernails
[37]	Healthy adults & children hair samples (*n* = 65)	Solvent extraction	LC-MS/MS	GHB, 0.11–0.96 ng/mg (mean, 0.38 ± 0.25 ng/mg, *n* = 44); GHB-GLUC, <LOQ (*n* = 3)
[40]	Athletes (*n* = 100) & sports students (*n* = 50) urine samples	Dilution	LC-QTOF-MS	GHB-SUL, 0.009–11.5 mgL; GHB-GLUC, 0.5–19.8 mgL

LC-MS/MS, liquid chromatography-tandem mass spectrometry; GC-MS/MS, gas chromatography-tandem mass spectrometry; GLUC, glucuronide; SUL, sulfate; ND, not detected; <LOQ, below the limit of quantification; LC-QTOF-MS, liquid chromatography-quadrupole-time-of-flight mass spectrometry.

**Table 3:** Summary of analysis of GHB-conjugates and other metabolites in human biological samples from GHB users.

Reference No.	Sample	Sample Preparation	Analytical Platform (Untargeted or Targeted)	Treatment (Administration Dose, Route, and No. of Doses)	Sampling Time	Metabolic Changes	Summary
[40]	Volunteers (*n* = 3), urine	Dilution	LC-QTOF-MS (untargeted)	2.25 g Xyrem^®^ (equal to 1.86 g of GHB) or 10 mL Somsanit (equal to 2 g of GHB)	Before and 72 h after administration	GHB-SUL (↑ but detected in non-GHB users), GHB-GLUC (fluctuate, lower than thresholds), GHB-GLUC/β-citryl glutamic acid ratio (↑)	Failure of GHB-SUL and GHB-GLUC as potent markers for GHB, GHB-GLUC/β-citryl glutamic acid ratio as a potential biomarker for GHB administration
[41]	Healthy men (*n* = 20), urine	Dilution	LC-QTOF-MS (untargeted)	50 mg/kg GHB (Xyrem^®^)	4.5 h after administration	Glycolate (↑), succinylcarnitine (↑), GHB-carnitine (↑), GHB-glutamate (↑), GHB-glycine (↑)	Discovery of new metabolites of GHB (GHB-carnitine, GHB-glutamate, and GHB-glycine)

LC-QTOF-MS, liquid chromatography-quadrupole-time-of-flight mass spectrometry; ↑, significantly increased vs. before administration.
